# The Living Environment and Thermal Behaviours of Older South Australians: A Multi-Focus Group Study

**DOI:** 10.3390/ijerph16060935

**Published:** 2019-03-15

**Authors:** Joost van Hoof, Helen Bennetts, Alana Hansen, Jan K. Kazak, Veronica Soebarto

**Affiliations:** 1 Faculty of Social Work & Education, The Hague University of Applied Sciences, Johanna Westerdijkplein 75, 2521 EN Den Haag, The Netherlands; 2 Department of Spatial Economy, Faculty of Environmental Engineering and Geodesy, Wrocław University of Environmental and Life Sciences, ul. Grunwaldzka 55, 50-357 Wrocław, Poland; jan.kazak@upwr.edu.pl; 3 School of Architecture and Built Environment, The University of Adelaide, North Terrace, Adelaide, SA 5005, Australia; helen.bennetts@adelaide.edu.au (H.B.); veronica.soebarto@adelaide.edu.au (V.S.); 4 School of Public Health, The University of Adelaide, North Terrace, Adelaide, SA 5005, Australia; alana.hansen@adelaide.edu.au

**Keywords:** seniors, older adults, temperature, elderly, housing, thermal sensation, thermal comfort, building services engineering, public health

## Abstract

Ageing brings about physiological changes that affect people’s thermal sensitivity and thermoregulation. The majority of older Australians prefer to age in place and modifications to the home environment are often required to accommodate the occupants as they age and possibly become frail. However, modifications to aid thermal comfort are not always considered. Using a qualitative approach this study aims to understand the thermal qualities of the existing living environment of older South Australians, their strategies for keeping cool in hot weather and warm in cold weather and to identify existing problems related to planning and house design, and the use of heating and cooling. Data were gathered via seven focus group sessions with 49 older people living in three climate zones in South Australia. The sessions yielded four main themes, namely ‘personal factors’, ‘feeling’, ‘knowing’ and ‘doing’. These themes can be used as a basis to develop information and guidelines for older people in dealing with hot and cold weather.

## 1. Introduction

As in other countries the population of Australia is ageing. In 2017 about 15% of the Australian population consisted of people aged 65 years or above [1] and in the next 40 years the proportion is expected to grow to 22% of the total population [2]. Ageing-in-place for as long and comfortably as possible is the preference of the majority of older Australians and the focus of government strategies [3,4]. Home modifications and assistive devices may be required to accommodate the occupants as they age and particularly when they become frail [5]. However, modifications to aid thermal comfort are not always considered [6,7,8]. 

As people age, they experience physiological changes that affect their thermal sensitivity and thermoregulation [8,9,10,11]. These changes include reduced vascular reactivity, lower metabolic rate, and reduced muscle strength. Hot and cold environments can further exacerbate age-related medical conditions, rendering older people vulnerable to both heat and cold-related mortality and morbidity [12]. Very hot weather can have a significant health impact on older people, with studies showing an increase in cardiovascular conditions and heat-related morbidity such as headache, shortness of breath, and heat stress during heat waves [13,14,15,16,17,18]. Likewise, living in cold conditions increases the chance of experiencing illnesses such as arthritis, influenza, pneumonia and asthma as well as blood pressure changes, cardiac conditions, and strokes, which can lead to death [12,19]. 

Air-conditioning is routinely proposed as a measure to offset the effects of extreme heat [20,21]. Most people also rely on space heating during winter to warm their homes. However, even when an air-conditioner or heater is installed, it may not be used or operated effectively by older residents. This may be due to concerns over inability to pay the rising energy prices [22,23], house design and layout [24], the design of the equipment [25], sensory factors, or cultural and attitudinal reasons [26]. Therefore, the underlying thermal performance of the dwelling itself is important.

Outside Australia, thermal comfort of older people has been widely studied [27,28,29,30,31,32], but most of the studies used quantitative techniques based on field studies and climate chamber tests. On the other hand, a qualitative approach to thermal comfort, as Hitchings notes [33], allows researchers to understand the cultural context of thermal aspects in daily lives.

In Australia, research into thermal comfort and the thermal environment in housing of older people has only recently emerged. In 2012 a small study was undertaken in a rural town in Victoria during a hot period to investigate the relationship between housing quality and the thermal comfort of older people [34]. The study found that the age of buildings as well as wall and roof constructions played an important role in determining the indoor thermal comfort. Earlier, a study by Williamson et al. [35] investigated the health benefits for vulnerable groups (including older adults) of improving the energy performance requirements for new housing. They suggested that minimising temperature extremes within buildings by increasing the energy-efficiency rating of the design may yield significant savings to Australia’s health sector. In Queensland, a study by Miller et al. [36] explored the relationship between the building construction and design, internal temperatures and electricity consumption of an aged care facility. The study identified the need for building regulations to improve the minimum standards of housing to improve occupant health and safety. 

Bills and Soebarto [37,38] investigated the thermal comfort of older people in 18 households in metropolitan Adelaide, South Australia. The study found that in summer, the indoor temperature range when these older people deemed their environment to be ‘slightly warm’ and ‘warm’ was similar to that when younger adults voted ‘neutral’ on the 7-scale of thermal comfort. In winter when the participants voted ‘cool’ or ‘cold’, almost 50% of the time they indicated that they did not want to be warmer although the minimum indoor temperature was as low as 12 °C. This seems to be in contrast with other studies, which showed that older people generally prefer a warmer environment than younger subjects [11,26]. 

The study reported in this paper focuses on gaining an in-depth understanding of the ways older people keep cool in hot weather or warm in cool weather, and any issues associated with achieving thermal comfort, heating and cooling equipment or house design. The study is part of a wider research project that aims to develop information for designers, care-givers and the general public about strategies for achieving indoor comfort with the minimum amount of energy in order to support the health and well-being of older Australians who are ageing-in-place. The wider research includes a survey of older people’s thermal behaviour as well as monitoring of the thermal environment of their homes and recording their thermal comfort experience, addressed in separate papers. 

## 2. Methodology

An interactive, qualitative study design was chosen for the current study, namely multiple focus group sessions. The qualitative methodology allows the researchers to listen to the lived-experiences of the participants, in order to develop an understanding of the interaction between the various factors affecting their thermal comfort and to explore the wider social and cultural context in which these factors occur [39,40]. The qualitative approach complements quantitative research techniques common in thermal comfort research [41] and, in this case, will influence forth-coming aspects of the broader research project. In the following paragraphs, the focus group as a method, ethics, the participants, the focus group procedure, and data analysis and management are described.

### 2.1. Focus Groups and Ethics

Focus groups are particularly useful for exploring people’s knowledge and experiences. The format can encourage participation from those who are reluctant to be interviewed on their own, and encourage contributions from people who feel they have nothing to say. This approach enables insights to be gained into the participants’ shared understanding of the issues. Focus groups are useful when exploring subjective meanings and understandings from the research participants’ perspective, and the similarities and differences in participants’ views and experiences [42,43,44,45]. The focus group methodology has been used in housing and technology implementation studies previously [46,47,48,49]. 

The study was conducted in accordance with the Declaration of Helsinki [50], and ethical approval was obtained from the Human Research Ethics Committee of the University of Adelaide (Approval number H-2018-042). 

### 2.2. Participants

**Figure 1:**
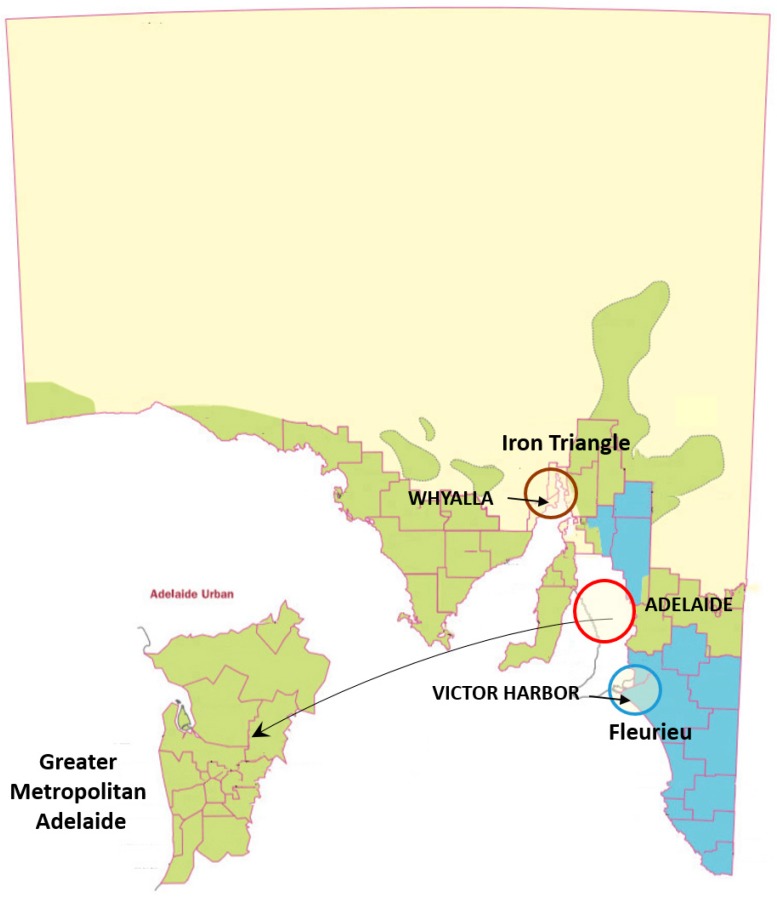
Climate zones in South Australia and the three study regions: Iron Triangle *BSk*; Adelaide *Csa*; Fleurieu *Csb* [51].

Participants were recruited from local government associations, community groups and through a press release picked up by local newspapers. The criteria for selection were that participants were 65 or older (although two somewhat younger participants were allowed to join) and were living independently in the community. There were no exclusion criteria, such as the type of housing (detached housing, apartment, terraced housing) people lived in, nor were there any limits set in terms of socio-economic status or the demand for care or level of dependency. Participants, however, had to be able to communicate in spoken English. The participants were drawn from three climate zones in the state of South Australia; hot dry (*BSk*), warm temperate (*Csa*) and cool temperate (*Csb*) according to the Köppen–Geiger climate classification system [51] (Figure 1). The climate of South Australia has distinct seasonal variations with hot dry summers (that often include heatwaves) and cool, wet winters. The diurnal range in both summer and winter can be large. *BSk* climate is found in the Iron Triangle area (Port Pirie, Port Augusta and Whyalla) and is a semi-arid or a steppe climate. *Csa* is found in the Greater Metropolitan Adelaide and is a hot-summer Mediterranean climate. *Csb* is found in the Adelaide Hills and the Fleurieu Peninsula, and is a warm-summer Mediterranean climate. 

A total of 49 participants—eight males and 41 females—took part in seven focus group sessions that were held in July and August 2018 (Table 1). Participants’ ages ranged from 61 to 98 with an average of 77. Most people lived in a single detached house (72%) and the rest in a semi-detached unit, townhouse or house. There were no apartment dwellers. Only two people were still in the paid workforce, 65% of the participants received a part or full pension and the remainder were self-funded retirees.

### 2.3. Procedure

All participants were provided with information about the research prior to the session and signed informed consent forms before the focus group began. The goal of the focus group was explained to the participants at the start of the session then an introductory text and code of conduct were read out. Every session was guided by a session leader and two assistants. All session leaders and assistants had been briefed about the procedure and had access to a printed instruction guide about how to run the session. The role for the session leader was to stimulate conversation and to obtain a maximum of variety in responses and input by prompting a number of questions (Table 2). The assistants’ role was to support the session leader and to take notes. During the focus group sessions conversations were audiotaped and later transcribed verbatim by a professional transcriber. Transcripts were anonymised to ensure confidentiality. 

### 2.4. Data Analysis and Data Management

Data from the focus groups were analysed using standard qualitative techniques [52]. All transcripts were read by the relevant researchers (the session leaders and assistants) and the analysis included a process of open, axial and selective coding. A thematic analysis was conducted to identify major themes with the assistance of NVivo software v12 (QSR International Pty Ltd., Doncaster, Victoria, Australia). Quotes that summarised the essence of the participants’ experiences were identified. Researcher triangulation was applied during the entire process, for example, separate analyses of the transcripts were conducted by two of the researchers who reached consensus over the findings and the emerging themes with the help of the third researcher. 

## 3. Results

The findings of the qualitative analyses provide a general understanding of the relationship between weather, older people’s behaviour and attitudes toward heating and cooling, well-being, and household energy use and aspects of their home’s planning and design. 

**Figure 2:**
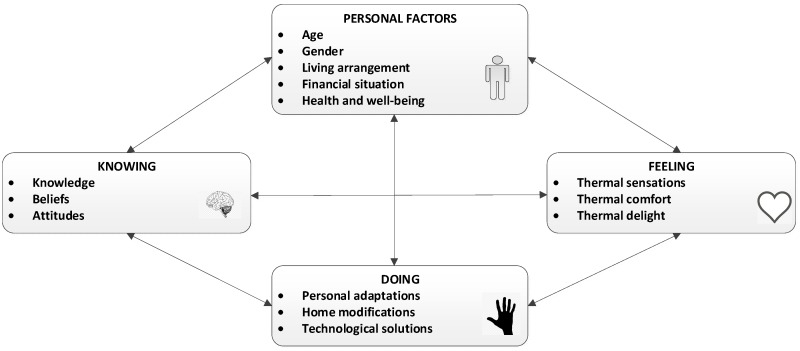
A human-centric model of how older South Australians deal with the thermal qualities of the living environment.

Data analysis resulted in the identification of four main themes representing how participants deal with the thermal qualities of the living environment. These themes are ‘personal factors’, ‘knowing’, ‘believing’ and ‘doing’, as represented in Figure 2. In the following paragraphs, these themes are described in detail. Relevant quotes from the focus group are presented to provide the context of the analysis.

### 3.1. Personal Factors

Comments from all participants clearly indicated that personal factors such as an individual’s age, living arrangements and socio-economic or financial situation influenced how they perceived their thermal environment and how they responded to hot and cold weather. 

Many participants reported that getting older had affected their perception of the thermal environment.

“As I’ve aged, I fear the cold and I feel the cold more than when I was younger. The heat doesn’t affect me as much, I haven’t noticed suffering from heat, but I do know that as I’ve become older, I suffer more from the cold weather.”

[Focus group 3 *Csa*]

“My body can’t adjust. When it’s hot I can’t take the heat and I feel so cold nowadays so I definitely feel a big change.”

[Focus group 5 *Csb*]

Many indicated that with ageing their activity levels and mobility were decreasing and this had implications for their thermal comfort.

“I think that in many cases we’re not as active as we used to be so we’re not warming ourselves up but we’ve also got a lot more time to think about it rather than when we had a job.”

[Focus group 5 *Csb*]

In some cases, people’s activity changed because they had a spouse with mobility impairment.

“I’m less active, and because [my husband]’s fine if he’s sitting or lying—and I like to keep him company and I also don’t like to go too far in case something happens—then I’m tied to the house too.”

[Focus group 1 *Csa*]

While many participants indicated that they now spent more time at home, one participant said that retirement brought more freedom to control the home’s thermal environment, in comparison with when this person was still at work and confined to the work space.

“I was a teacher for a very long time and I had to stay in the hot classroom regardless. Now when it’s hot I can do other things, be in a cool spot, put the fan on, turn the air cooling on, do nothing.”

[Focus group 2 *Csa*]

Many participants indicated a personal preference for either hot or cold weather. Some stated that their partner had different thermal preferences and, therefore, it was difficult to create thermal conditions that were optimal for both. Others just said they took the weather as it comes.

“But I much prefer the heat to the cold. I hate the cold, I hate winter.”

[Focus group 7 *BSk*]

“I don’t think it affects me at all. I just go with the weather.”

[Focus group 7 *BSk*]

### 3.2. Feeling

Sensory experience was revealed to be an important aspect of the thermal environment of the focus group participants. Expressions about heating and cooling were often connected not only to thermal sensations (such as feeling hot or cold) and comfort (such as feeling comfortable from experiencing moving air), but also and more importantly to thermal delight, for example the delight that comes from the warmth of sunshine. It is more about the mood affected by the presence of the sun or the wind rather than simply about experiencing heat and cold or air movement.

“If it’s a very, very drizzly typical winter’s day I think that your whole attitude is in the doldrums, you’re really “I don’t feel like going out today”, whereas if you get out of bed of a morning, beautiful blue sky and there’s no breeze blowing even if it’s in the middle of winter it’s just gorgeous to work out in the garden or somewhere or sit out in the front patio. I think the weather has got a lot to do with the way you feel.”

[Focus group 1 *Csa*]

“It can be fairly cold but if the sun is out, I feel better.”

[Focus group 5 *Csb*]

“[On] sunny days I feel a lot better. It just seems to help make my mood a little bit better. […] It’s always affected me, if it’s a sunny bright day it just feels better.”

[Focus group 5 *Csb*]

“If you have moveable air; it’s somewhat of a relief.”

[Focus group 3 *Csa*]

### 3.3. Knowing 

The theme of ‘knowing’ concerns the way that the participants’ knowledge, beliefs and attitudes influenced how they understood and dealt with the thermal environment. The knowledge might be based on past experience or learned knowledge. Their environmental and financial beliefs and attitudes also influenced their actions in dealing with their thermal environment.

#### 3.3.1. Knowledge Based on Past Experience

Some people had experiences of living in other climate zones in Australia with more extreme weather, such as tropical northern Queensland, the outback of Western Australia or the wet, cold regions of northern Victoria, while others had grown up or spent time living in England or Canada. These experiences had provided knowledge of how to cope with particular sorts of weather.

“We had the good luck to go over to Canada and Alaska and someone made the comment, “It’s not how cold you are in Alaska; it’s how many layers of clothing you’ve got on”, so you just dressed accordingly. That was a little bit of a clue to say how to do things.”

[Focus group 1 *Csa*]

A vast majority of the participants grew up in a time when there were fewer heating and cooling technologies available; air-conditioning for cooling was almost unknown and heating was often provided by open fires or simple electric radiators. Nonetheless participants indicated that their childhood experiences provided knowledge that one could survive without air-conditioning and also provided a repertoire of passive techniques.

“I can remember we had linoleum floors and during a heatwave in the summer we would like on the linoleum floors to play Monopoly or whatever it was.”

[Focus group 3 *Csa*]

“[W]e used to put an ice block in front of the fan and where I sometimes go and stay in the country, we still do that, we take a block of ice and put it in front of the fan and you’d be amazed how cooling that is.”

[Focus group 3 *Csa*]

#### 3.3.2. Health and Well-Being

A number of participants had physical disabilities which affected their mobility. During the course of the discussions several people referred to their health issues—both physical and psychological—and many revealed particular beliefs about the links between their health or well-being and the weather.

“We’re very fortunate to have around this time of an afternoon a terrific sunlight coming into our lounge and we will sit in there sometimes because of the arthritis to get the warmth.”

[Focus group 1 *Csa*]

“[F]rom observing my wife she certainly gets grumpier, it has affected her mood and her attitude in the hot weather. In the cold she’s fine but if it’s a really hot day she gets quite niggly, if you know what I mean?”

[Focus group 6 *Csb*]

A few people indicated they had chronic health problems or were taking medication. They connected cold weather with experiencing aches and pains, chest problems and arthritis and said that cold weather aggravated symptoms of arthritis. This in turn caused stress and frustration because they could not do many things. While most mentions of health issues related to problems in cold weather, one person noted that her asthma symptoms worsened in hot weather.

“Arthritis is an issue. Chronic pain is an issue. [These issues] are intensified by extremes in weather.”

[Focus group 2 *Csa*]

“Yeah, I need to be inside, locked in by about half past three. I don’t go out in the evenings in the winter time at all. […] Just the change of temperature affects your chest.”

[Focus group 7 *BSk*]

“My asthma is worse in summer when it’s hot and windy and there’s dust in the air and lots of seeds, much more than in winter. It’s the hot winds and particularly if there’s a fire anywhere and the smoke, but particularly the hot dirty winds with dusty winds we get, that affects my health quite dramatically.”

[Focus group 3 *Csa*]

“Well I suffer from asthma so I have to be very careful. I have to rug up and put a scarf on so I have to be very careful in the cold weather.”

[Focus group 4 *Csa*]

#### 3.3.3. Learned Knowledge

Some participants revealed a good knowledge about passive design strategies such as building orientation and materials.

“The positioning of the house makes a big difference. And one of my sons […], they’ve got big windows in their house and they never get sun on their windows in the summer. Yeah, the position makes a big difference.”

[Focus group 5 *Csb*]

“We have a south-facing house, the whole width of the house at the front is a south-facing window and in winter that’s the room that we have the gas heater in. All we do—it’s not double glazed—we pull a full a curtain and it goes right across and it gives that insulation.”

[Focus group 1 *Csa*]

Many participants exhibited knowledge about their local climate including the seasonal and diurnal variations while others revealed beliefs that were not always based on official weather information.

“At night-time it generally cools down more in Adelaide than other places.”

[Focus group 1 *Csa*]

“And if you check the temperature it’s usually one or two degrees warmer in winter and cooler in the summer.”

[Focus group 6 *Csb*]

“If we get a hot north wind you get the heat but that hasn’t happened very often. Sometimes you’ll get two or even three days of a hot north wind, we have in the past, but this last summer we haven’t had much.”

[Focus group 6 *Csb*]

It is worth noting that many participants in the hot dry climate (*BSk*) believed that in summer the outdoor temperature was up to 10 °C higher than in Adelaide even though the real difference, according to the data from the Bureau of Meteorology, was only around 3 °C. This belief no doubt affected the participants’ perception.

#### 3.3.4. Beliefs and Attitudes

Participants’ environmental beliefs and attitudes indicated an understanding of the connection between electricity generation, energy use and heating and cooling technologies. In some cases, this had led to people installing solar panels or adopting passive techniques.

“I’m made conscious of it by the vast quantity of power that we consume and so our strategy for dealing with that is to put in an extensive solar system.”

[Focus group 6 *Csb*]

“I’m just concerned about the environmental, of generating so much electricity and power.”

[Focus group 3 *Csa*]

Another participant indicated the opposite attitude to the link between environmental concerns, electricity generation and heating and cooling.

“I think was it the Paris Agreement where they talked about climate change and the use of coal? Since they’ve stopped using coal generating electricity the prices have gone up. The others are subsidised. We’re paying a lot for a political decision. […] I just flick a switch and it sorts my problems.”

[Focus group 2 *Csa*]

The cost of energy was also discussed in all the focus group sessions. There had been considerable media coverage about rising energy costs in the period preceding the focus groups. Many examples of beliefs about energy costs appeared to be influenced by this coverage. All participants were concerned about rising energy costs. For those receiving a pension, paying energy bills was a major concern. Even those participants who were financially better off said that the thought of potential energy bills influenced their actions.

“But yeah it scares me because I know my electricity bill is due any day now and I have paid some off of it but I’m still scared of how much it’s going to cost me because we’re on a pension, to come up with the money to pay it off is just—and we’re the highest State, well we’re the highest in the world aren’t we in electricity costs?”

[Focus group 4 *Csa*]

“Aren’t we about the 5th most expensive country in the world for electricity?”

[Focus group 1 *Csa*]

“South Australia I believe has the dearest electricity in the world, and I for one can’t afford to pay it. Every three months I shudder when I take it out the letterbox.”

[Focus group 2 *Csa*]

There was a range of attitudes about whether or not heating and cooling was essential or a luxury. Many of those who saw it as essential compared the cost of heating and cooling to other expenses such as buying cigarettes, the cost of a couple of cups of coffee or the cost of ill-health.

“$10 a day is a lot cheaper than if he was hospitalised or had any consequences from being just too cold”.

[Focus group 1 *Csa*]

“I won’t go without my air-conditioner, I can’t, otherwise I’ll finish up in the [hospital] like I had before with the heat. My concern is not finishing up in the [hospital] so I have my heater on or my cooler on, that’s my first priority.”

[Focus group 4 *Csb*]

In fact, for most people, it is difficult to know how much their heating and cooling energy use contributes to their total energy bills. Only one person identified this issue.

“Well it’s very hard to know how expensive each particular item is unless you’ve got separate meters to each of the units, and I think if you really want to know, that’s about the only way to go.”

[Focus group 5 *Csb*]

Some participants seemed to suggest that they thought their energy bill was entirely due to their use of heating and cooling.

“It costs too much. My winter electricity bill was up around $700 or $800 and I only have it on when I need to, it’s terrible.”

[Focus group 7 *BSk*]

### 3.4. Doing

The discussions indicated that participants’ personal factors, feeling and knowing influenced their actions in dealing with cold and hot weather. When they discussed how they kept warm in cold weather and cool in hot weather, they referred to a variety of personal practices as well as issues to do with the house and with technology. 

#### 3.4.1. Personal Adaptive Strategies

Participants referred to a broad range of personal adaptive strategies including changing their diet and clothing according to the seasons with lighter food and lighter clothing in hot weather and heavier food and layers of warm clothes, particularly of natural fibres in colder weather.

“I certainly do change our diet slightly for more warming food, [my husband] makes the soup and we have soup for lunch. I usually will do a casserole or two, warmer food but salads in the summer.”

[Focus group 3 *Csa*]

“For me, I’ve always hated the cold, all my life so it’s always been dress more, dress more and dress more.”

[Focus group 1 *Csa*]

Other strategies included using rugs and blankets in cold weather, particularly when sitting still for long periods watching TV or reading and taking hot showers (in cold weather) or cool showers (in hot weather).

“I’ve got a crocheted blanket my daughter made me and I’ve got that underneath and if I want that bit extra on my legs, well, I just wrap that around me and I’m quite happy there without any heat on.”

[Focus group 6 *Csb*]

“I love a wheat bag to warm up what you’re getting into, it’s just lovely. It gets cooler but you’ve warmed up. I just love my wheat bag. It’s much nicer than a hot water bag because it doesn’t make a noise.”

[Focus group 1 *Csa*]

“I rely a lot on my bath, a hot bath in winter and a cold bath in summer before going to bed, and during the day in the summer time, if I feel I’m getting hotter I get into the bath, I keep the water in there.”

[Focus group 5 *Csb*]

“I was going to say that I’ve got all the rugs on and then my cat is on my lap.”

[Focus group 2 *Csa*]

Some of the more unusual strategies for dealing with very hot weather involved one person sitting with her feet in a tub of cold water or another who said she kept a dress in the freezer.

“The way I keep cool is into the freezer, I’ve got a shelf in the freezer that I have two dresses during the summer and one goes in the freezer and one of them I put on. After an hour or so I’ll change them. I know that I’ve always got something cold. When my air-conditioner broke down that was a godsend.”

[Focus group 7 *BSk*]

Many participants tried to be as active as possible during winter.

“Go outside, do something outside. […] Come back inside and you’ll feel warm, work in the garden.”

[Focus group 6 *Csb*]

“Yes, I think exercise is the main thing in winter, particularly. If I exercise first thing in the morning I find that that helps me right through the day because then your body is already warm.”

[Focus group 2 *Csa*]

One participant said that when it was very cold, he spent about 11 hours in bed as a strategy to keep warm. Other people mentioned changing the timing of activities, particularly during very hot weather when they might stay indoors during the heat of the day and go out first thing or after the sun went down.

“Because of daylight saving and because of the climate you can do your gardening at six in the morning or 7:30 at night.”

[Focus group 1 *Csa*]

“Yes, I get up early, do what I need to, close the house up, sit and do whatever sewing craft work that I want to do until the sun goes down at night.”

[Focus group 4 *Csa*]

In extreme heat many participants said they would go to public air-conditioned venues such as libraries or shopping centres. This was not only to ‘borrow’ the cool air but to also avoid the feeling of ‘cabin fever’ associated with being in a confined space. Others mentioned going out to a relative’s house as a way to avoid the cost of air-conditioning.

“I’m more likely to go to the library than to go shopping but it’s the same thing, you go there and read the paper and it’s nice and cool.”

[Focus group 2 *Csa*]

“The shops are inside and then it’s all air-conditioned, so you get away from that trapped feeling of being in one room in one small house.”

[Focus group 2 *Csa*]

“I know lots of people around me that will go to a son or daughter’s house for the day, shut down their house for the day and go and have the two of them warmer in another house than in 2 separate houses.”

[Focus group 2 *Csa*]

For those who could afford it, another option was to holiday in a warmer climate during the very cold weather.

“I find the winter makes me keen to go north.”

[Focus group 6 *Csb*]

“People […] take off from here and go up to Queensland. A lot of people do.”

[Focus group 6 *Csb*]

#### 3.4.2. Changes to Home

In addition to personal adaptive strategies, participants manipulated or used the architectural design of the home, adjacent buildings and landscaping to deal with hot and cold weather and stay comfortable. In some cases, where the participants had built their own home, they were able to incorporate passive design principles such as good levels of insulation and appropriate orientation and shading.

“That’s why ours were built—this was a kit home developed in Queensland to cope with cyclones, so it’s a slab floor and it faces north with these great big windows so in winter if you’ve got your curtains open the slab heats up but in summer because the sun is higher and it’s built with metre wide eaves so they’re really wide, so you can go outside in the afternoon and have a look and the sun really doesn’t touch the walls but it does in winter.”

[Focus group 5 *Csb*]

“We had the chance when we built our house 20 years ago, we had extra insulation in the walls, we had extra insulation in the ceiling and we didn’t have to have big air-conditioning.”

[Focus group 3 *Csa*]

A number of participants referred to changes they had made to existing houses to improve their thermal performance.

“Ours has got insulation and we put cladding on the outside of the house about 7 or 8 years ago and that’s got insulation in under the cladding so it’s really good, it helps to keep the temperature down a bit, that helps as well.”

[Focus group 7 *BSk*]

“We had the windows put in and they’re the ones that when you slide them up from the bottom they slide down from the top and it’s made a big, big, big difference.”

[Focus group 1 *Csa*]

And I’ve done things like put polar fleece on all the windows as a lining, and put pelmets above all the windows so that that room is really efficient, put carpets on the floor, make sure that it’s an insulated area that’s being heated or cooled.”

[Focus group 2 *Csa*]

The situation was very different however for the participants living in social housing. They had much less scope to make changes to the house itself. Many of these homes had limited insulation, heating and cooling was not provided and it could be difficult and time-consuming to arrange maintenance.

“I think they need more insulation in their homes, but the [housing authority] don’t do much.”

[Focus group 7 *BSk*]

“I’m with [the housing authority] and of course they don’t supply anything, they don’t supply heating or cooling.”

[Focus group 4 *Csa*]

“It’s old houses and sometimes I think you get to a certain age and they think to themselves, “Oh well you’ll soon be out of there, you haven’t got much longer to go”, exactly. That’s the attitude, they don’t care, they don’t care.”

[Focus group 4 *Csa*]

Many participants referred to ‘operating’ the house in various ways—opening curtains to admit solar gain on colder days, shutting external or internal blinds, curtains or shutters in hot weather to reduce solar gain, or opening windows and doors to let cooling evening breezes into the house after a hot day. They also referred to minimising the area they heated or cooled by ensuring doors were shut.

“I keep the house shut and the blinds down and do all of that. I don’t have a problem with the heat unless I have to go out in it.”

[Focus group 6 *Csb*]

“In the summertime I’ll zone cool one area of the house. And in the winter time I’ll zone heat one area of the house because I’m lucky enough to have a full house system and I’ll just take on the lounge room.”

[Focus group 2 *Csa*]

“The other thing I would be saying is that I’m of an age where we understand some of this stuff. Some of the younger ones we know don’t know how to open windows, it’s got to do with turning the heating and cooling on and off so I guess I’m concerned about that as an issue as well. It’s the day and night thing, summer and winter, the same thing in the winter we open in the middle of the day to let the hotter air in so we do quite a bit.”

[Focus group 1 *Csa*]

#### 3.4.3. Technological Solutions

Participants discussed both personal devices and space heaters and coolers that they used to stay comfortable in the South Australian climates. 

##### Personal Devices

Many participants used personal heating devices such as electric throws in cold weather. One person also referred to a personal cooling device.

“It’s called warming the person as compared to warming the zone and what I do at home, I’ve got [a chair] and I’ve got a woolen under-blanket which is probably something you’re not familiar with, and then I have a single blanket heating pad on there and then I’m on it and then I’ve got a blanket over top of me and I’ll sit and watch TV.”

[Focus group 2 *Csa*]

“It’s called an electric throw, it’s like an electric blanket and I thought when they bought it—because they knew I get hot and I thought, “Why would they do that?” and I tried it one night and I tell you what, I’ve had it for 2 years and I use it every day. It’s absolutely glorious, and you don’t have to put the heater on and it’s a lot cheaper to do that than what it is to use the heater.”

[Focus group 4 *Csa*]

“If it’s really hot I’ve got a little personal air-conditioner thing, a little battery with a little bit of water in it and a little fan, just put it by you and it makes you cool.”

[Focus group 6 *Csb*]

##### Heaters

Participants referred to a number of different forms of space heating—gas, electric under-floor, wood fires, and reverse-cycle (heat pump) systems that provided both heating and cooling. Some had heating ducted throughout the house while in other cases the heating was localised to a room, (often the main living area) or else was portable. There were few comments about preferences or problems with heating systems. A couple of people said they preferred radiant sources of heat such as a wood fire. One participant mentioned problems with setting the thermostat herself, as the print was too small for her to read and someone else commented that it would be difficult for someone with arthritis to turn a gas heater on.

“Previously in my previous houses I’ve always used a wood heater, always saying that by the time you’ve chopped the wood you don’t need the heat but by the same token that is to us the most comfortable heat of all.”

[Focus group 5 *Csb*]

“I’ve got reverse cycle air-conditioning and I do put it on in the kitchen living area, close all the doors, close everything up, and I might put it on for one hour in the morning and that’s enough to warm, then I don’t put it on until dinner time because usually I’m active so I’m not sitting inside, and it does use power I agree but it is actually very effective.”

[Focus group 3 *Csa*]

“We’ve got those two heating lights and we turn them on—you get out of bed in the morning and before you have a shower you turn it on for three or four minutes.”

[Focus group 1 *Csa*]

“I like when you’re older, underfloor heating because if your feet are warm the rest of you tends to be warm.”

[Focus group 5 *Csb*]

##### Coolers

Most of the participants had either reverse-cycle (heat pump) or evaporative cooling, either ducted to the whole house or a split-system servicing individual areas. Reverse cycle systems appeared to be more common as they can provide heating as well as the cooling however some participants preferred evaporative cooling saying it was cheaper to run and better suited to the dry heat of South Australia’s summer. 

Interestingly, not all participants used cooling systems in summer with some saying either they had a cooler but it did not work or that they rarely felt the need to use one.

“[W]e only have evaporative air-conditioning. We’ve got two units one on the back half of the house and one on the front. […] I find in Adelaide’s dry climate evaporative air-conditioning, and it doesn’t take much to run compared with the refrigerated air-conditioning, it’s just so cheap.”

[Focus group 1 *Csa*]

“I’m in a retirement village and we’ve just got a split system because that’s what was there and I find the cooling is really good if it gets really hot.”

[Focus group 5 *Csb*]

“I bought an air-conditioner when I was in the other house. But since I’ve been in this one. I think I’ve used it once, that’s all. Not for me but for somebody else that came, but I don’t use it.”

[Focus group 4 *Csa*]

“I don’t like that cold air from the air-conditioner, I have arthritis and that causes me more problems than I need.”

[Focus group 2 *Csa*]

##### Fans

Many participants had ceiling fans, and they were widely used as an alternative to the cooling systems particularly in bedrooms.

“We’ve got overhead fans […] They are brilliant.”

[Focus group 1 *Csa*]

“If I ever built a house I would put [ceiling fans] in every room because I just believe that they suit my body. I don’t like that cold air from the air-conditioner.”

[Focus group 2 *Csa*]

“The best investment I’ve ever done is to have ceiling fans in all the rooms. I don’t have to use my air-conditioning very much at all during summer I just use my ceiling fans, unless I get visitors again then I’ll put it the air-conditioner on.”

[Focus group 4 *Csa*]

“I think from memory it was only once and it was the 10-day heatwave in Adelaide that we actually had the air-conditioner going at night, the rest was ceiling fans.”

[Focus group 5 *Csb*]

## 4. Discussion

The focus group discussions allowed the many elements influencing individual heating and cooling behaviour to be explored. This study builds on the results of a telephone survey on thermal conditions as experience by older South Australians by Soebarto et al. [53], and provided a more in-depth analysis of how older South Australians deal with both cold and warm conditions. Thermal comfort of older people is a widely studied domain, which is often based on quantitative approaches [8,30]. The findings of this study build on the foundations of thermal comfort research that explores the less quantifiable aspects of the thermal environment, as illustrated by the work of Heschong [54]. Heschong described the “thermal delight” in buildings, and wrote that “*the thermal environment […] has the potential for such sensuality, cultural roles, and symbolism that need not, indeed should not, be designed out of existence in the name of a thermally neutral world*” ([54], p. 17). 

This qualitative study identified a set of themes that reflect the way people interact with the home and deal with the way it responds to outdoor climatic conditions, and which are illustrated by a rich set of examples from daily living of older South Australians.

In all three of the South Australian climate zones where the focus groups were held, the four themes—personal factors, feeling, knowing and doing—always came to the fore during the discussions. The current study revealed that the strategies that individuals employed to keep cool in hot weather and warm in cold weather were complex, inter-related and influenced by a range of issues including personal factors and preferences, people’s beliefs and experiences, the type and design features of the dwelling, the type of heating and cooling equipment, as well as their financial concerns, regardless of the home’s location. 

There were some commonalities among the strategies. The participants discussed using a range of personal adaptive strategies before turning on the heater or cooler, which is also described by the theory of adaptive thermal comfort [55]. For some people this was because of their environmental concerns, for others it was for financial reasons or because it was just what they had always done. There was a strong sense that the knowledge of how to keep warm or cool without using heaters or coolers was valuable although it was either not known or not appreciated by many younger people. Concerns over the energy cost for heating and cooling were also shared among all participants, although in some cases this was expressed in terms of concern not for themselves but for other people who were older and/or struggling financially. 

There appeared to be some differences in ‘knowing’ and ‘doing’ associated with the age of the participants that warrant further exploration. There was an indication of increased use of air-conditioning by the oldest participants (85 years and over) and that this was related to two factors. First, age was connected to reduced mobility, and as a result, for example, it may have been harder to operate blinds or to exercise, or leave the home to go to a cool space like a shopping centre. It would be much easier to simply turn on the air-conditioner. Second, age was connected to decreasing health, and for some participants this condition often required a constant thermal environment which could only be achieved by turning on the air-conditioner.

By contrast, many of the younger participants (60–70) appeared to have good knowledge about and the ability to control their thermal environment. Investigating risk factors associated with heat-related health impacts, Belanger et al. [56] found an age-related increase in risks up to the age of 64 but these declined for those aged 65 or more. The authors suggested that while thermal perceptions may be reduced for the older people in their study this age group may be less at risk than the younger participants because they may not have to leave their dwelling for work or other commitments. 

Some differences in the ‘knowing’, ‘feeling’ and ‘doing’ were noted between the group living in the *BSk* and *Csb* climate zones. Those living in the former climate tended to believe that the climate in their area was much hotter than other areas in South Australia whereas those living in the latter climate believed that their environment was much cooler than the other areas. As a result, they took different strategies to deal with the weather. For example, more varieties of personal strategies to keep cool were mentioned in the focus group discussion in the *BSk* than in the *Csb* location.

While older people are often considered as one group in society, this study indicated that they are very heterogeneous, with a diverse range of circumstances and abilities. This means that in future quantitative work on older people and thermal comfort, a distinction should be made for age (younger seniors/older seniors), housing type (single family home/multifamily), or financial means (disposable income level, ownership status of the home) and health status. In addition, future work should also try to include more male participants in the sample. This heterogeneity of older people, in turn, has implications for thermal comfort strategies and advice. For example, information will need to be different for an older person who:

- feels the heat badly and has severe mobility issues and difficulty leaving the home, or;
- someone who rents a house that was built decades ago with little concern for the climate, or;
- a person who is still quite active with strong environmental convictions and who is better placed financially.

This matches calls for individual approaches when it comes to thermal comfort and older people by other researchers including, van Hoof et al. [57], Hansen et al. [58], and Hansen and Bi [59]. Similarly, a range of information about appropriate house design is required. Strategies for sustainable housing for older people are emerging all over the world [60,61] and may help improve the situation of older people in relationship to fuel poverty to varying degrees. 

Issues that concern people, buildings, energy use and environmental questions are complex often requiring a multi-disciplinary approach to research. Wierzbicka et al. [62] highlight the need for researchers to have a holistic awareness of the problem to ensure assumptions are considered in their wider context and to avoid measures that may improve one aspect of the problem but impact other aspects.

One factor that was not discussed in the current study was the impact of dementia on how older people cope with the indoor environment, thermal conditions and the weather. None of the participants mentioned this as a personal problem nor said that they were taking care of family members or spouses who had dementia. Overall, the level of dependency or the need for care was low among the participants of this study. In a future study, a sample comprising more frail older people should be included to better reflect the full spectrum of seniors, including people with dementia and mild cognitive impairment. It may, however, be challenging to include them in regular focus group discussions. Nevertheless, future work needs to consider the impact of dementia because as people age, the possibility of developing dementia increases, and dementia has a strong impact on how people are able to live independently and cope with the challenges of daily living [63,64,65]. 

It needs to be stressed that the studied population were living independently, rather than in an institutional setting such as a nursing home or and assisted-living facility. There are many studies available on the optimal design of the thermal environment of nursing homes and retirement villages, from both Australia [66,67] and from Europe [68]. In general, the populations of these facilities are in poorer health and often deal with the consequences of dementia [69,70], and, therefore, have fewer adaptive behavioural strategies at their disposal.

A broad range of information about thermal comfort for ageing-in-place is, therefore, necessary: information about adaptive strategies, energy efficient house design and modifications, using heaters and coolers efficiently and appropriately, as well as possible technology adaptations. 

## 5. Conclusions

A qualitative study to understand the way older South Australians keep warm in cold weather, cool in hot weather and issues relating to the planning and house design of the existing living environment has been conducted through focus group discussions involving 49 older people living in three climate zones. The study found that the issues were complex and there was no one single answer to understanding the thermal behaviour of older people. Three main themes were identified as things that have to be first understood and considered: ‘personal factors’, ‘feeling’ and ‘knowing’, as these influenced what participants did to deal with the weather—the ‘doing’, which is the fourth theme. The extent to which each strategy was implemented varied and depended on the individuals’ personal factors, feelings they would like to have, and their belief, attitudes and knowledge about the weather and their living environment. Nevertheless, personal adaptive strategies were almost always undertaken first despite the fact that some design changes to their house had been implemented or technological solutions had been or would be implemented. 

The study suggests that the four themes identified be used as a basis to develop information and guidelines for older people in dealing with hot and cold weather. There is no one-size-fits-all strategy as there is a wide range of conditions and circumstances around older people that affect their needs. What is common among all is a concern over the energy cost from running air-conditioning systems—heaters or coolers. Design guidelines as well advice to older people to cope with the weather should therefore address this cost concern while understanding personal factors, the feeling and sensation older people want to have in their home environment, as well as their belief and knowledge, need to be carefully considered.

## Figures and Tables

**Table 1:** Description of the study participants.

Group	Date	Number of Participants and Sex	Average Age (Range) [years]	Location, Climate Zone
1	13 July 2018	*n* = 2 males *n* = 4 females	73 (65–83)	Unley, *Csa*
2	17 July 2018	*n* = 1 male *n* = 6 females	77 (69–84)	Campbelltown, *Csa*
3	20 July 2018	*n* = 2 males *n* = 6 females	76 (63–84) *	Unley, *Csa*
4	25 July 2018	*n* = 6 females	76 (61–88) *	Playford, *Csa*
5	2 August 2018	*n* = 1 male *n* = 9 females	79 (67–88)	Woodside, *Csb*
6	8 August 2018	*n* = 2 males *n* = 3 females	74 (71–87)	Victor Harbor, *Csb*
7	15 August 2018	*n* = 7 females	85 (74–98)	Whyalla, *BSk*
Total		49	77	

* One participant in this group was younger than 65.

**Table 2:** Prompts during the focus group.

1. How do you think age affects how you feel when it is hot or cold? Do you do things differently now to keep warm/cool—than, say, when you were in your 30s–40s?
2. How do you think the weather affects your health or well-being? What about the impact of temperatures or conditions inside the house? What about the impact on your sleep? On your stress level?
3. How do you keep yourself warm in cold weather? Depending on the answer, then ask: when, how long, why operate it that way? Do you avoid getting cold or do you take action once you feel cold?
4. How do you keep yourself cool in hot weather? Depending on the answer, then ask: when, how long, why operate it that way? Do you avoid getting hot or do you take action once you feel hot?
5. If you had the opportunity to do so, what changes would you make to your current house to make the temperature more comfortable during hot or cold weather? And if you were able to build a new house, what would you do differently from the house you live in now?
6. And what about the heaters and coolers themselves? Do you have issues with them? [Prompt only when necessary: remote control; thermostat issues; noise/vibration; smell; usability; safety concerns/risk; environmental concerns; running costs].
7. There has been a lot of new coverage about electricity costs. Do concerns about costs have an impact on you in terms of using your heater or cooler?
8. We are interested in your ideas about the best ways of providing information for older people about things like—How to keep warm and why it is important?
